# TGFβ1 secreted by cancer-associated fibroblasts induces epithelial-mesenchymal transition of bladder cancer cells through lncRNA-ZEB2NAT

**DOI:** 10.1038/srep11924

**Published:** 2015-07-08

**Authors:** Junlong Zhuang, Qun Lu, Bing Shen, Xiaojing Huang, Lan Shen, Xi Zheng, Ruimin Huang, Jun Yan, Hongqian Guo

**Affiliations:** 1 Nanjing Drum Tower Hospital, Nanjing University Medical School, Nanjing, Jiangsu 210008, China; 2 Department of Urology, Shanghai First People’s Hospital, Shanghai Jiaotong University, Shanghai, 200080, China; 3 Model Animal Research Center, MOE Key Laboratory Model Animal for Disease Study, Nanjing University, Nanjing, Jiangsu 210061, China; 4 Department of Radiology, Memorial Sloan Kettering Cancer Center, New York, NY 10065, USA; 5 Nanjing Urology Research Center, Nanjing, Jiangsu 210008, China

## Abstract

Urinary bladder cancer (UBC) patients at muscle invasive stage have poor clinical outcome, due to high propensity for metastasis. Cancer-associated fibroblasts (CAFs), one of the principal constituents of the tumor stroma, play an important role in tumor development. However, it is unclear whether CAFs from UBC induce cell invasion and which signaling pathway is involved. Herein, we found that conditional medium from UBC CAFs (CAF-CM) enhanced the invasion of UBC cells. CAF-CM induced the epithelial-mesenchymal transition (EMT) by regulating expression levels of EMT-associated markers in UBC cells. Higher concentration of TGFβ1 in CAF-CM, comparing with the CM from adjacent normal fibroblast, led to phosphorylation of Smad2 in UBC cells. Additionally, inhibition of TGFβ1 signaling decreased the EMT-associated gene expression, and cancer cell invasion. Interestingly, a long non-coding RNA, ZEB2NAT, was demonstrated to be essential for this TGFβ1-dependent process. ZEB2NAT depletion reversed CAF-CM-induced EMT and invasion of cancer cells, as well as reduced the ZEB2 protein level. Consistently, TGFβ1 mRNA expression is positively correlated with ZEB2NAT transcript and ZEB2 protein levels in human bladder cancer specimens. Our data revealed a novel mechanism that CAFs induces EMT and invasion of human UBC cells through the TGFβ1-ZEB2NAT-ZEB2 axis.

Urinary bladder cancer (UBC) is one of the most common malignancies worldwide, with 74,690 estimated cases in USA during 2014^1^. The estimated mortality of bladder cancer is 15,580 cases in USA, which has not changed much within last ten years^1^. There are roughly two biologically different transitional cell carcinomas of bladder cancer. One is papillary pathway with the mutation of H-Ras and FGFR3, and the other is muscle invasive pathway with frequent p53 and RB mutations. The latter is correlated with poor prognosis. Importantly, about 15% of papillary bladder cancer patients will eventually develop into muscle invasive type^2^. Epithelial–mesenchymal transition (EMT) is frequently observed in invasion fronts of neoplastic cancer cells with the polarized shape, which is considered as one of the major risk factors for metastasis^3^,^4^,^5^. However, few studies have elucidated clearly how bladder cancer cells with different biological characteristics were induced to metastasis. Therefore, it is urgent to fully understand the common molecular mechanisms underlying bladder cancer development.

The initiation and progression of tumor are complicated biological processes, with multiple gene mutations in a stepwise manner in epithelial cells^6^. Recently, tumor stroma is also demonstrated to influence the aggressiveness and drug resistance of cancer cells^7^,^8^,^9^. Cancer-associated fibroblast (CAF) is one of the major components in the tumor stroma, which plays a critical role in tumor growth and angiogenesis^10^. Through secreting various cytokines, CAFs stimulate cancer cell growth and invasiveness. In line with this, the expression levels of CAF markers, such as FSP1 and FAP, have been used to predict clinical outcomes in multiple cancer types^11^. However, the molecular mechanisms how CAFs regulate bladder cancer cell aggressiveness, particularly, how CAFs regulate the EMT in bladder cancer, are not well-known.

Long noncoding RNAs (lncRNAs) are a group of noncoding RNAs with the length longer than 200 nucleotides. They have been shown to be involved in various biological processes, including tumor development^12^.The regulation of lncRNAs in response to extracelluar stimuli may increase cancer cell migration and invasion capacities. Recent study also demonstrated that lncRNA-ATB is induced by long-term TGFβ1 treatment, promoting liver cancer cell migration and invasion^13^. Another lncRNA MALAT1, induced by TGFβ1, is overexpressd in UBC samples, essential for cancer cell metastasis^14^,^15^. These findings support lncRNAs as essential players mediate the extracellular stimuli and cancer cell behavior.

Currently, few studies focused on whether and how CAFs modulate bladder cancer cell aggressiveness through lncRNAs. In this study, we have investigated whether CAFs induce bladder cancer cell EMT and invasiveness through paracrine effect. We also reported that lncRNA-ZEB2NAT mediates bladder cancer cell invasion, which is induced by TGFβ1. Finally, we examined their clinical correlations in human bladder cancer specimens.

## Results

### Characterization of primary NFs and CAFs

**Figure 1:**
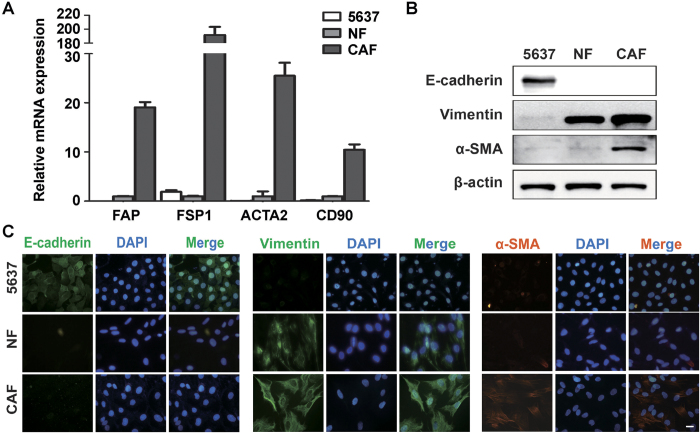
Characterization of primary cultured NFs and CAFs. **A**: The mRNA expression levels of CAF-specific genes, including FAP, FSP1, ACTA2 and CD90, in 5637 cells (an epithelial cell control), NFs and CAFs by qRT-PCR using β-actin gene as the normalization control. **B**: The protein expression levels of E-Cadherein, Vimentin and α-SMA in 5637 cells, NFs and CAFs were detected by immunoblotting. **C**: Immunofluorescence staining showed the subcellular location and the expression of E-cadherin, Vimentin and α-SMA in 5637 cells, NFs and CAFs. Scale bar, 10 μM.

The CAFs and NFs were isolated from three bladder tumor tissues and adjacent normal bladder mucosae. In order to test the purity of CAFs and NFs, we examined fibroblast biomarkers in these cells. As shown in Fig. 1A and Suppl. Fig. 1A, mRNA expression levels of four CAF-specific genes, including fibroblast activation protein (FAP), fibroblast specific protein 1 (FSP1), alpha-smooth muscle actin (ACTA2) and CD90, were significantly increased in CAFs, compared to NFs and UBC 5637 cells (an epithelial cell control). Western blotting assay showed that: 1) epithelial cell marker (E-cadherin) was only detected in 5637 bladder cancer cells; 2) mesenchymal cell marker (Vimentin) was highly expressed in both NFs and CAFs; and 3) myofibroblast marker (α-SMA) was overexpressed only in CAFs (Fig. 1B and Suppl. Fig. 1B). Immunocytochemistry staining further confirmed that primary cultured fibroblast populations (NFs and CAFs) only express Vimentin, but not E-Cadherin. α-SMA expression was higher in CAFs than NF and 5637 cells (Fig. 1C). Altogether, these data indicated that we successfully isolated CAFs with high purity from bladder cancer specimen.

### Conditional medium from CAFs (CAF-CM) induced bladder cancer cell migration and invasion

**Figure 2:**
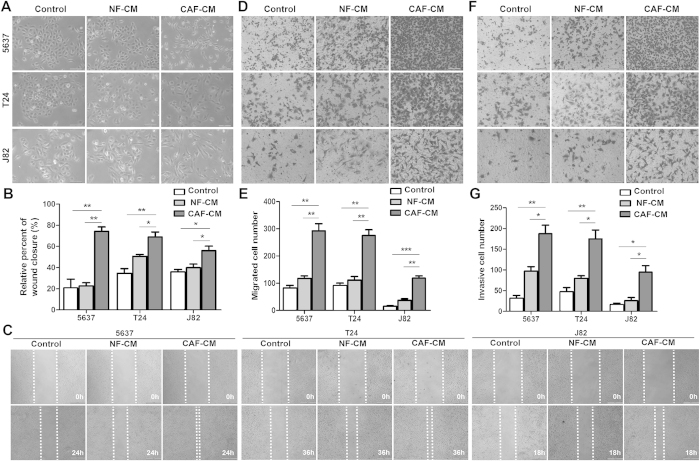
CAF-CM increased the cell migration and invasion capabilities of bladder cancer cell lines. **A**: Morphological features of bladder cancer cell lines under culture medium, NF-CM and CAF-CM, respectively. **B**,**C**: Cell migration ability was measured by a wound-healing assay. The relative percent of wound closure was calculated at 24 h in 5637 cells, 36 h in T27 cells, and 18 h in J82 cells, respectively, before the complete would closure. **D**–**G**: Cell migration (**D**,**E**) and cell invasion (**F**,**G**) were measured by the Transwell cell migration/invasion assay. *P < 0.05, **P < 0.01, ***P < 0.001.

In order to assess whether CAF-CM can increase bladder cancer cell motility, we treated three different human bladder cancer cells (5637, T24 and J82) with culture medium, NF-CM and CAF-CM. Treatment with CAF-CM strikingly induced morphological changes in all of three cancer cells, including fewer cell junctions, elongated pseudopodia in scattered cells and more spindle-like shapes (Fig. 2A and Suppl. Fig. 1C,D). Wound healing assay was performed to examine whether CMs from CAF and NF could affect the cell migration rate. As Fig. 2B,C shown, NF-CM had no significant effect on wound healing time in comparison with control medium. However, CAF-CM treatment greatly accelerated cell migration rates, 3.5 folds in 5637 cells, 2.0 folds in T24 cells and 1.5 folds in J82 cells, compared with control medium (Fig. 2B,C). Transwell assay was then used to evaluate both cell migration and cell invasion. CAF-CM increased cell migration more than two-folds in all three bladder cancer cell, which is consistent with the results of wound healing assay (Fig. 2D,E). Moreover, CAF-CM stimulation also extremely raised the numbers of invaded cells attaching on bottom chamber (Fig. 2F,G, Suppl. Fig. 1E–H). Above three different assays indicated that some molecules secreted by CAFs into the CAF-CM may induce bladder cancer cell migration and invasion in a paracrine manner.

### CAF-CM induced EMT in three UBC cell lines

**Figure 3:**
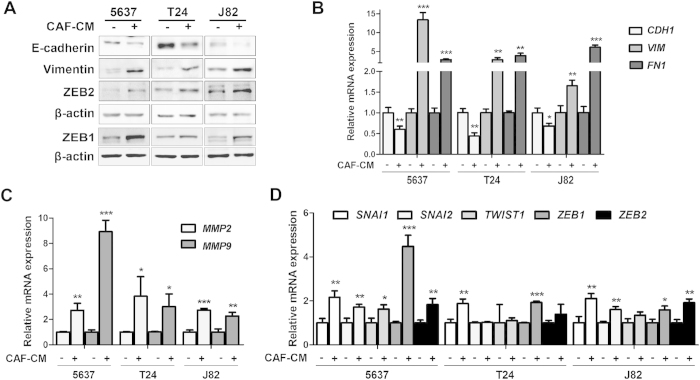
CAF-CM induced EMT phenotypes in three bladder cancer cell lines. **A**: The protein expression levels of E-cadherin, Vimentin, ZEB1 and ZEB2 in the CAF-CM treated bladder cancer cell lines by immunoblotting. β-actin protein was used as the loading control. **B**: The mRNA expression levels of epithelial marker (CDH1, encoding E-Cadherin), mesenchymal markers (VIM and FN1) (**B**) invasion markers (MMP2 and MMP9) (**C**) and EMT-associated transcription factors (**D**) in the CAF-CM treated bladder cancer cell lines by qRT-PCR using β-actin gene as the normalization control. *P < 0.05, **P < 0.01, ***P < 0.001.

EMT has been reported to be related to cancer cell invasion and metastasis. Since morphological changes were observed upon CAF-CM treatment (Fig. 2A and Suppl. Fig. 1C,D), we investigated whether these cell shape changes were EMT by testing the expression levels of EMT-associated genes. Western blotting analysis (Fig. 3A and Suppl. Fig. 1I) showed that in three different epithelial bladder cancer cell lines, the treatment with all of three CAF-CMs led to the decrease of E-Cadherin expression (epithelial cell marker) along with the increase of Vimentin (mesenchymal cell marker), ZEB1 and ZEB2 expression (EMT-associated transcription factor). Quantitative RT-PCR verified that CDH1 gene, encoding for E-Cadherin, was suppressed at mRNA level; whereas two mesenchymal markers (vimentin and fibronectin) were significantly up-regulated in CAF-CM-treated 5637, T24 and J82 cells (Fig. 3B). In addition, invasion related MMP-2 and -9 genes also increased in CAF-CM-treatment group (Fig. 3C). Furthermore, mRNA levels of transcription factors regulating EMT, such as SNAI1, SNAI2, TWIST1, ZEB1 and ZEB2, were also analyzed by quantitative RT-PCR (Fig. 3D). Overexpression of SNAI1 and ZEB1 transcripts were detected in all three bladder cancer cell lines stimulated with CAF-CM. These data implied that EMT was a potential mechanism for cell migration and invasion induced by CAF-CM.

### Paracrine effect of TGFβ1 in CAF-CM

**Figure 4:**
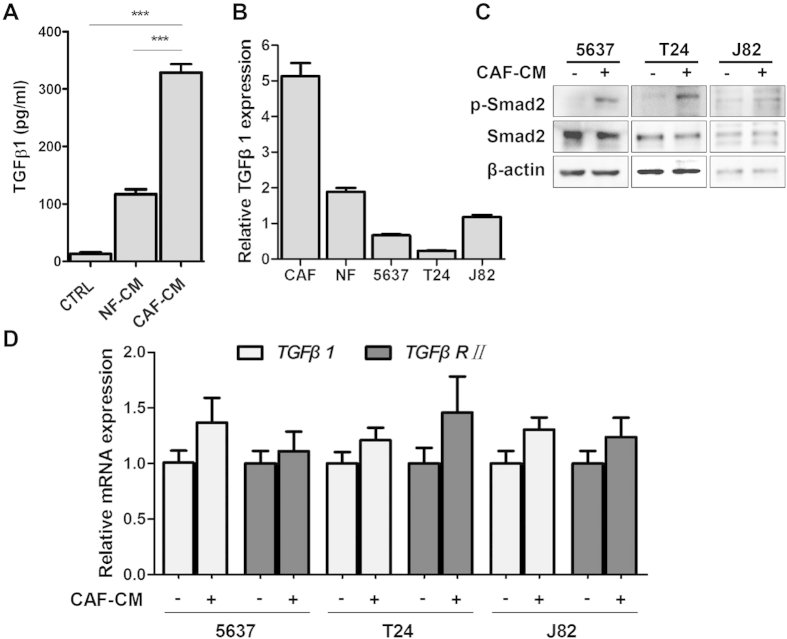
CAFs secreted TGFβ1 to activate TGFβ/Smad signaling in bladder cancer cells. **A**: TGFβ1 in conditional mediums secreted by 5637 (CTRL), NF and CAF cells were quantified by ELISA. **B**: The mRNA expression levels of TGFβ1 in CAFs, NFs and three bladder cancer cell lines by qRT-PCR using β-actin gene as the normalization control. **C**: The expression of phosphorylated Smad2 and total Smad2 protein in the CAF-CM treated bladder cancer cell lines by immunoblotting. β-actin protein was used as the loading control. **D**: The expression levels of TGFβ1 and TGFβRII in bladder cancer cell lines cultured with CAF-CM were detected by qRT-PCR using β-actin gene as the normalization control. ***P < 0.001.

In order to identify which CAFs-secreting cytokine may induce EMT and invasion of bladder cancer cells, we compared TGFβ1, TGFβ2 and TGFβ3 mRNA levels in NFs versus CAFs, and found that TGFβ1 was the most highly expressed cytokine in CAFs than NFs (Suppl. Fig. 2). We also examined the CAF-CM, NF-CM and condition medium from 5637 cells (CTRL) using a cytokine ELISA and higher level of TGFβ1 was detected in the CAF-CM (328.8 pg/ml) than those in the NF-CM (116.9 pg/ml) and CTRL (12.6 pg/ml; Fig. 4A). ELISA data also confirmed larger amount of TGFβ1 in CMs from another two CAFs, compared with that in their NF-CMs (Suppl. Fig. 3A). qRT-PCR also confirmed that TGFβ1 mRNA transcripts were the most abundant in CAFs, about 2.7, 7.7, 22.6 and 4.3-fold the amount in NFs, 5637, T24 and J82 cells, respectively (Fig. 4B). Downstream targets of TGFβ1 signaling in three bladder cancer cell lines under CAF-CM stimulation were further tested. Western blotting assay showed that Smad2 was phosphorylated but the total Smad2 expression remained unchanged (Fig. 4C), indicating the activation of canonical TGFβ signaling under the CAF-CM treatment.

To demonstrate the activation of TGFβ signaling in epithelial bladder cancer cells is mainly triggered by the TGFβ1 secreted by the CAFs (paracrine activation), the expression levels of TGFβ1 and TGFβRII in 5637, T24 and J82 cells, with and without CAF-CM treatment, were assessed by qRT-PCR. As Fig. 4D shown, neither of TGFβ1 nor TGFβRII expression was significantly changed in CAF-CM treatment group, implying that TGFβ signaling activation induced by CAF-CM was not due to autocrine or reverse-paracrine mechanisms.

**Figure 5:**
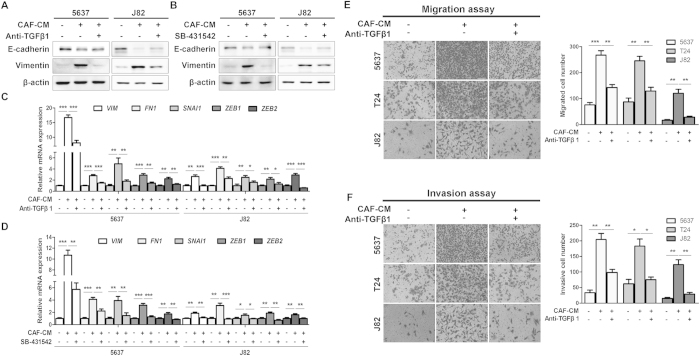
EMT phenotypes were reversed by blocking TGFβ/Smad signaling in the CAF-CM treated bladder cancer cells. **A**,**B**: The protein levels of E-cadherin and Vimentin in the CAF-CM treated 5637 and J82 cells upon the blocking of a neutralizing antibody (**A**) or a TGFβR1 inhibitor, SB-431542, (**B**) by immunoblotting. **C**,**D**: The mRNA levels of mesenchymal markers and EMT-associated transcription factors in the CAF-CM treated 5637 and J82 cells upon the blocking of a neutralizing antibody (**C**) or a TGFβR1 inhibitor (SB-431542) (**D**) by qRT-PCR. Cell migration (**E**) and invasion ability (**F**) in the CAF-CM treated 5637, T24 and J82 cells upon the blocking of a neutralizing antibody by the Transwell cell migration/invasion assay. *P < 0.05, **P < 0.01, ***P < 0.001.

Paracrine activation of TGFβ1 by CAF-CM was further confirmed by the TGFβ1 blocking assays using a neutralizing TGFβ1 antibody or a TGFβRI small molecule inhibitor (SB-431542). Western blotting showed both abrogation of TGFβ1 binding to its cognate receptor (Fig. 5A) or inhibition of TGFβ signaling pathway (Fig. 5B) suppressed the CAF-CM-induced Vimentin expression and slightly increased the expression of E-Cadherin in 5637 and J82 cells. The results were confirmed in another two pairs of CAFs/NFs, when blockade of TGFβ1 signaling by SB-431542 (Suppl. Fig. 3B,C). Consistently, TGFβ1 neutralizing antibody or SB-431542 suppressed the CAF-CM effects on expression of the EMT-associated genes, including Vimentin (VIM), Fibronectin (FN1), SNAI1, ZEB1 and ZEB2, at mRNA level (Fig. 5C,D). In addition, the CAF-CM induced cell migration (Fig. 5E) and invasion (Fig. 5F) could also be inhibited by the TGFβ1 neutralizing antibody in 5637, T24 and J82 cells. The inhibitory effects on invasiveness were also confirmed in another two pairs of CAFs/NFs, when blockade of TGFβ1 signaling by SB-431542 (Suppl. Fig. 3D–G). Moreover, the treatment of three UBC cell lines with TGFβ1 for 48 h induced cancer cell invasion (Suppl. Fig. 4A,B) and EMT associated protein levels (Suppl. Fig. 4C), indicating that TGFβ1 alone is sufficient to induce EMT in UBC cell lines tested.

### TGFβ1 in CAF-CM induced cell invasion partially through increased ZEB2NAT lncRNA-ZEB2 transcription factor axis in bladder cancer cells

**Figure 6 f6:**
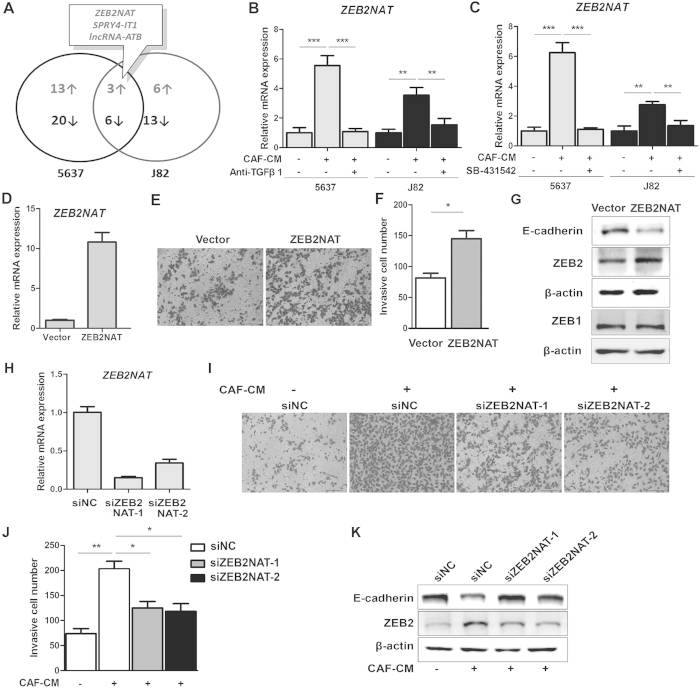
TGFβ1 in CAF-CM induced cell invasion partially through ZEB2NAT lncRNA-ZEB2 transcription factor axis in bladder cancer cells. **A**: Summary of altered expression of lncRNAs in the CAF-CM-treated 5637 and J82 cells, comparing to the NF-CM-treated ones. 1.5-fold of relative mRNA level using qRT-PCR, normalized by β-actin gene, was used as the threshold for significant changes. **B** and **C**: ZEB2NAT expression levels in the CAF-CM treated 5637 and J82 cells upon the treatments of a TGFβ1 neutralizing antibody (**B**) and a TGFβRI inhibitor (SB-431542) (**C**), respectively. **D**: Exogenous expression of ZEB2NAT lncRNA in 5637 cells, detected by qRT-PCR using β-actin gene as the normalization control. **E**–**G**: Effects of ZEB2NAT lncRNAs overexpression on cell invasion by the Transwell invasion assay (**E**,**F**) and protein levels of E-cadherin, ZEB1 and ZEB2 by immunoblotting (**G**) in 5637 cells. H: Knockdown of ZEB2NAT by RNAi using two siRNAs targeting different regions of the lncRNA (siZEB2NAT-1 and siZEB2NAT-2) in 5637 cells. The relative ZEB2NAT expression levels were at 48 hours after siRNA transfection and determined by qRT-PCR using β-actin gene as the normalization control. **I**,**J**,**K**: Effects of ZEB2NAT lncRNAs knockdown on cell invasion by the Transwell invasion assay (**I**,**J**) and protein levels of E-cadherin and ZEB2 by immunoblotting (**K**) in the CAF-CM treated 5637 cells. *P < 0.05, **P < 0.01, ***P < 0.001.

Cumulative evidences support that lncRNAs are involved in multiple stages of cancer development. To dissect how CAF-CM regulates lncRNAs, along with the EMT-associated genes, we performed PCR array to examine 72 cancer-related lncRNAs (Suppl. Fig. 5A). We validated that lncRNA PCR array in several cell lines and only single RT-PCR product was observed for each set of primers (Suppl. Fig. 5B). Up-regulation of 3 lncRNAs and down-regulation of 6 lncRNAs by 1.5-fold change were identified in both 5637 and J82 cells treated with CAF-CM (Fig. 6A; Supplementary Table S1). Notably, ZEB2NAT, a natural antisense transcript to ZEB2 gene, was among the up-related lncRNAs. To verify whether ZEB2NAT is regulated by TGFβ1 in CAF-CM, we used the neutralizing TGFβ1 antibody (Fig. 6B) and SB-431542 inhibitor (Fig. 6C) to block TGFβ1 signaling and found that both treatments decreased the CAF-CM-induced ZEB2NAT expression in 5637 and J82 cells. The inhibitory effects on CAF-CM induced ZEB2NAT expression were also confirmed in another two pairs of CAFs/NFs, when blockade of TGFβ1 signaling by SB-431542 (Suppl. Fig. 3H,I). Consistently, treatment of TGF β1 induces ZEB2NAT transcript in both of these two UBC cells (Suppl. Fig. 4D). Overexpression of ZEB2NAT was then established in 5637 cells (Fig. 6D). Forced expression of ZEB2NAT induced UBC cell invasion about 1.5 folds, comparing the empty vector-transfected cells (Vector) (Fig. 6E,F). Moreover, ZEB2NAT overexpression slightly increased ZEB2 protein and repressed E-Cadherin protein levels, without change of ZEB1 protein level (Fig. 6G). Moreover, to examine whether ZEB2NAT is involved in CAF-CM induced EMT and cell invasion, on the other hand, RNA interference, involving two siRNAs targeting different regions, was employed to knockdown ZEB2NAT expression. The knockdown efficiency was achieved more than 65% by either of siZEB2NAT RNA (Fig. 6H). Transwell invasion assay revealed that depletion of ZEB2NAT significantly reduced the CAF-CM-induced cell invasion (Fig. 6I[Fig f6]J). Western blotting data further demonstrated that ZEB2 protein levels were reduced in two ZEB2NAT knockdown groups, while E-Cadherin protein levels were increased (Fig. 6K).

### Positive correlation of TGFβ1, ZEB2 and ZEB2NAT in UBC specimens

**Figure 7:**
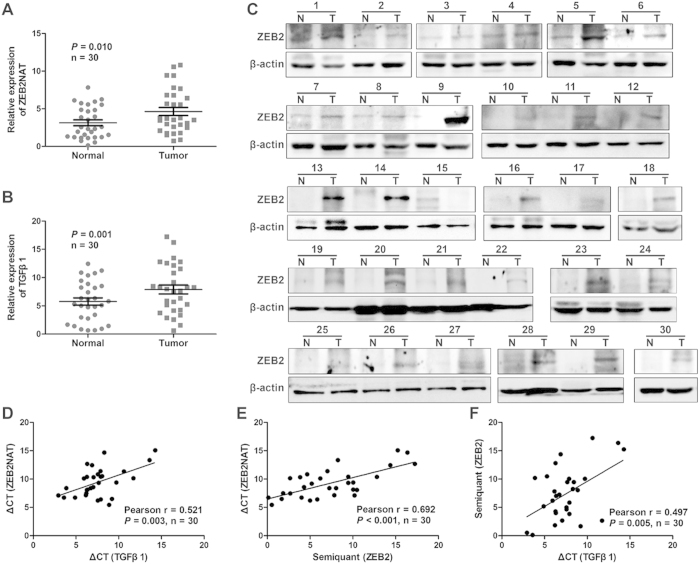
Correlations of TGFβ1, ZEB2NAT, and ZEB2 in clinical UBC samples. The expression levels of ZEB2NAT (**A**) and TGFβ1 transcripts (**B**), and ZEB2 protein (**C**) in 30 human bladder cancer tissues (Tumor or T) and the paired normal tissues (Normal or N) from the same patient. Relative ZEB2NAT and TGFβ1 mRNA levels were assessed by qRT-PCR and normalized by β-actin gene. **D**–**F**: The correlations among ZEB2NAT, TGFβ1 and ZEB2 were decided by Pearson correlation analysis. ΔCT values in qRT-PCR were used as the expression levels of ZEB2NAT and TGFβ1 transcripts. ZEB2 protein bands on Western blot were quantified by Image J software. *P* < 0.05 was considered statistically significant.

The correlations among TGFβ1, ZEB2 and ZEB2NAT were investigated in 30 human UBC samples. Quantitative RT-PCR revealed that ZEB2NAT and TGFβ1 are significantly increased by 1.5 folds (*P* = 0.010) and 1.4 folds (*P* = 0.001) in human UBC samples, respectively (Fig. 7A,B). Western blotting data also demonstrated that ZEB2 protein level significantly increased in 26 out of 30 human UBC samples (Fig. 7C). Interestingly, we observed the significant increased levels of TGFβ1 mRNA (2.3 folds, *P* = 0.019), ZEB2NAT transcript (2.12 folds, *P* = 0.026) and ZEB2 protein (1.45 folds, *P* = 0.012) in muscle invasive group (MI), compared to non-muscle invasive group (NMI; Suppl Fig. 6). The correlations between the mRNA levels of ZEB2NAT and TGFβ1 by qRT-PCR assay plus the ZEB2 protein levels by Western blotting assay were also analyzed by Pearson’s correlation analysis. ZEB2NAT mRNA level was highly correlated with TGFβ1 mRNA level (r = 0.521, *P* = 0.003, Fig. 7D) and ZEB2 protein level (r = 0.692, *P* < 0.001, Fig. 7E). TGFβ1 mRNA level is also associated with ZEB2 protein level (r = 0.497, *P* = 0.005, Fig. 7F). These data indicate the high incidence of activation of TGFβ1-ZEB2NAT-ZEB2 axis in human bladder cancer patients, rendering high migration and invasion capabilities to the bladder cancer cells.

## Discussion

Emerging evidences support that tumor stroma is actively involved in cancer development^16^. CAFs are one of the most abundant cells in tumor stroma, providing a supportive microenvironment for and induce more aggressive behaviors of cancer cells^17^,^18^,^19^,^20^. Patients with MIBC usually have poor prognosis, with high propensity for metastasis^21^,^22^. In this study, we confirmed that CAFs at MIBC stage have typical myofibroblast characteristics, with the expression of α-SMA, FAP, FSP and CD90^23^,^24^, whereas very low or undetectable expression levels in NFs. We are the first to prove that CAFs from MIBC patient induce migration and invasion of three bladder cancer cells with different characteristics. CAF-CM can prominently induce EMT in epithelial-like bladder cancer cells (5637 and T24) or promote EMT in mesenchymal-like bladder cancer cells (J82), indicating there exist paracrine effects on bladder cancer cells. The tumor supportive effects of CAFs have also been identified in different cancer types, such as breast cancer, prostate cancer, and gastric cancer^25^,^26^,^27^. Taken together, the induction of EMT in cancer cells by CAFs in the tumor stroma is a common mechanism underlying the acquisition of metastatic potential of cancer cells.

CAFs around the cancer regions are not only able to support cancer cell growth but also to promote invasion and metastasis through the secretion of cytokines and inflammatory mediators. For example, uPA secreted by CAFs as an activator of matrix-degrading protease can cleave pro-MMPs to upregulate MMPs activity, which contributes to angiogenesis and metastasis^28^,^29^. In addition to remodeling ECM, CAFs also secrete various cytokines, such as FSP1 and hepatocyte growth factor, to induce tumor metastasis, which are not found in normal fibroblasts^30^,^31^. In ovarian cancer, downregulation of miR-214 in CAFs leads to increase chemokine CCL5 production and secretion into tumor microenvironment^32^. In our study, we found that CAFs express and secrete TGFβ1 at a higher level than NF. Secreted TGFβ1 induces EMT and cancer cell invasion in all of three bladder cancer cell lines. Notably, treatment of neutralizing TGFβ1 or TGFβR1 inhibitor, SB431542 reversed CAF-CM induced cancer cell invasion and EMT. We did not find the induction of TGFβ1 or TGFβR1 expression in cancer cells under CAF-CM treatment, further supporting the notion that TGFβ1 secreted by CAFs is an important factor to induce bladder cancer cell invasion.

TGFβ1 is a cancer-promoting factor for cancer progression, regulating a battery of target genes involved in EMT and metastasis^33^,^34^. In our study, CAF-CM activated canonical TGFβ signaling pathway through phosphorylating Smad2 in 5637 and J82 cells. Moreover, EMT-associated transcription factors, such as Snail and ZEB2 were also induced in CAF-CM treatment group, whereas their expression levels were abrogated at various degrees by pretreatment with neutralizing TGFβ1 antibody or TGFβR1 inhibitor.

In addition to coding genes, lncRNAs, shown as a new dimension for biological processes^35^,^36^,^37^, may also be regulated by TGFβ1. Our data successfully identified three lncRNAs (lncRNA-ATB, SPRY4-IT1 and ZEB2NAT) were upregulated in both of two UBC cell lines, 5637 and J82. LncRNA-ATB has been identified as overexpressed transcript in liver cancer cells with long term TGFβ1 treatment. Overexpression of lncRNA-ATB promotes liver cancer cell metastasis through inducing EMT and invasion. Mechanistically, lncRNA-ATB induces ZEB1 and ZEB2 through competitive binding with and blocking the function of miR-200 family. Concomitantly, it also induces IL11 expression, triggering STAT3 signaling^38^. Another lncRNA, SPRY4-IT1, has been implicated in melanoma, esophageal squamous cell carcinoma and renal cancer progression^39^,^40^. Overexpression of SPRY4-IT1 promotes melanoma cell proliferation and invasion, at least through regulation of lipogenesis^41^. Therefore, using the PCR microarray, we successfully identified lncRNAs related with cancer cell invasion.

ZEB2, one of the major transcription factors involved in EMT, directly represses E-cadherin during EMT^42^. Our data showed that CAF-CM induces ZEB2 in 5637 and J82 cells at mRNA (Fig. 3D) and protein levels (Fig. 3A). However, in T24 cells the induction of ZEB2 is not significant at mRNA level (Fig. 3D), whereas the ZEB2 protein level is induced by CAF-CM, suggesting that the regulation of ZEB2 in T24 cells may be through transcription-independent approach (Fig. 3D). Noncoding RNAs has been implicated in post-transcriptional regulation. Therefore, by screening 72 lncRNAs, we found that lncRNA ZEB2NAT is upregulated by CAF-CM, whereas such upregulation is abrogated by the pretreatment of neutralizing TGFβ1 antibody or TGFβR1 inhibitor, indicating that ZEB2NAT is regulated by TGFβ1. ZEB2NAT is a conserved natural antisense transcript corresponding to the 5′UTR of ZEB2^42^. The regulation of ZEB2 by ZEB2NAT is through prevention of the processing of a large intron located in ZEB2 5′UTR, resulting in keeping an internal ribosome entry site (IRES) for translation of ZEB2 protein^43^. In out study, knockdown of ZEB2NAT reversed CAF-CM induced ZEB2 protein level and partially inhibits the CAF-CM induced cancer cell invasion, indicating that ZEB2NAT is one of the essential TGFβ1 downstream components involved in EMT and cancer cell invasion. Overall, there exist at least two regulatory mechanisms to regulate ZEB2 expression: one is at transcriptional level and the other is at posttranscriptional level by lncRNA ZEB2NAT. The clinical association of TGFβ1, ZEB2NAT transcripts and ZEB2 protein were further confirmed in 17 human bladder cancer samples.

Given that upregulation of EMT-associated transcription factors (*eg*. SNAIL and ZEB2), and EMT-associated lncRNAs (lncRNA-ATB and ZEB2NAT) by CAF-CM, CAF-CM induces a full EMT reprogramming in bladder cancer cells at multiple levels. Consistently, overexpression of ZEB2 and Snail protein levels are negative associated with bladder cancer and predicts poor clinical outcome^43^,^44^,^45^,^46^. Taken together, these data further indicate that TGFβ1 induces EMT and invasion through a complicated regulatory mechanism at multiple levels. Moreover, targeting TGFβ1 pathway may be of value for UBC therapy, especially for MIBC patients.

## Materials and Methods

### Cell lines, transfection and human samples

Human bladder cancer cell lines, T24, 5637 and J82 were obtained from Cell Bank of Type Culture Collection, Chinese Academy of Science (Shanghai, China). Cells were maintained in RPMI1640 supplemented with 10% FBS, 100 units/ml penicillin and 100 μg/ml streptomycin at 37 °C. Bladder cancer cells were treated with the CM from NFs and CAFs, as well as an equal volume of complete culture medium. 50 μg/ml TGFβ1 blocking antibody (R&D Systems, MAB240, Minneapolis, MN, USA) was added to the media to neutralize TGFβ1, whereas 20 nM SB431542 (Selleckchem, S1067, Boston, MA, USA) was added 1 h prior to conditional medium treatment to block TGFβ signaling in bladder cancer cells. UBC cells were also treated with 2 nM TGF β1 for 48 h. For RNA interfering, two siRNAs (Genepharma, Shanghai, China) targeting ZEB2NAT (5′-CAGAAAUGGUGAGAAGAAAtt-3′ and 5′-GAACAGUUUUGGCCAGAAAtt-3′), as well as negative control (5′-UUCUCCGAACGUGUCACGUtt-3′), were transfected into cells using Lipofectamine® 3000 Reagent (Invitrogen, Carlsbad, CA, USA), according to the manufacturer’s instructions, respectively. For gene overexpression, ZEB2NAT expression plasmid was constructed in vector pcDNA3.1 (Invitrogen) using the PCR primers: Forward: 5′-GGGGTACCCTCAATAAAACTTTTCCTGGGCT-3′ and Reverse: 5′-TGCTCTAGAACAAAGATAGGTGGCGCGTG-3′. Both empty vector and ZEB2NAT overexpressed plasmids were transfected into cells using Lipofectamine® 3000. mRNA and protein were collected 48 h after transfection.

Human bladder cancer specimens and adjacent normal tissues, which are 3 cm far away from cancer lesions, were obtained from Drum Tower Hospital affiliated to Nanjing University. The protocols were approved by the Ethics Committee of Drum Tower Hospital for tissue sample collection and informed consent was obtained from all subjects. The methods were carried out in accordance with the approved guidelines. The bladder cancer patients had not treated with radiation therapy or chemotherapy before surgery. The bladder cancer specimen used for isolation of stromal fibroblasts was diagnosed as muscle invasive bladder cancer with histological grade II. The H&E staining and immunohistochemical staining for E-Cadherin and α-SMA confirmed that CAFs were surrounding the cancer nests. The fresh specimens were cut into small pieces and digested with 160 μg/ml collagenase I (Sigma, C9891, St. Louis, MO, USA) and 25 μg/ml hyaluronidase (Sigma, H4272) at 37 °C for 2 h. The mixture was strained through strainer (BD Biosciences, San Jose, CA, USA). Then the cells were collected and cultured in DMEM/F12 supplemented with 10% FBS, 100 units/ml penicillin and 100 μg/ml streptomycin. After 2–3 passages, a confluent and homogeneous monolayer of stromal fibroblasts was formed. In order to prepare conditioned medium of cultured CAFs and NFs, the normal fibroblasts and cancer-associated fibroblasts were cultured for 48 h and then the medium was collected and centrifuged for 10 min at 3,000 rpm to remove cell debris. All the fibroblasts used in the experiments were at less than 10 passages.

### Wound healing assay

Cells were seeded in 6-well plates with 5 × 10^5^ cells per well and cultured with different mediums. Then, a wound was made by using a 100 μl pipette tip on cell monolayer and photographs were taken at appropriate time to estimate the area occupied by migratory cells.

### Transwell assay

Transwell (Costar, New York, NY, USA) were used to evaluate the invasion and migration capacities of UBC cells *in vitro*. After treating with different media, 1 × 10^5^ cells in 500 μl serum-free medium were inoculated in the upper chamber, coated with (invasion assay) or without (migration assay) Growth factor reduced Matrigel® , and medium containing 10% FBS was added into the lower chamber as a chemoattractant. After incubation for the appropriate time, cells on the upper surface of the membrane were removed by wiping with Q-tip, and the invaded cells were fixed with formaldehyde and stained using 0.5% crystal violet (Sigma). The numbers of invaded and migrated cells were counted in five randomized high power fields under a microscope.

### RNA isolation and quantitative reverse transcription-quantitative PCR (qRT-PCR)

Total RNAs were extracted using TRIzol® (Invitrogen, 15596018) as manufactures’ instruction. Reverse transcription was conducted by using random primers in Takara system (Dalian, China). The expression of relative genes were measured by qRT-PCR using SYBR Green in an ABI 7500 StepOne Plus Real Time PCR instrument (Applied Biosystem, USA). The expression of target genes were calculated based on the cycle threshold (Ct) values comparative with a reference geneusing formula 2^−ΔΔCt^. β-actin was used as an internal control. qRT-PCR was performed in triplicate for each sample in a 10 μl reaction mixture, which was consisted of template cDNA (0.2 μl), primers (0.4 μl, l.0 M), ROX Reference Dye II (0.2 μl), dH_2_O (4.2 μl) and SYBR Premix Ex Taq (5 μl, SYBR® Premix Ex Taq Kit, Takara). Primer sequences used in our study were listed in Supplementary Table S2. As for lncRNA profiling, we used Human lncRNA Discover PCR array/TGFβ pathway (Bio-Serve Company, BS-lncRNA002, Shanghai, China), which consisting of 72 lncRNAs, derived from the lncRNA database (www.lncRNAdb.org) and several positive genes which are regulated by TGFβ, such as Vimentin. Total RNA was isolated from J82 and 5637 cells treated with and without CAF-CM for 48 h, respectively. The values were normalized by the β-actin, which was the internal control. ΔΔCt values (with CAF-CM versus without CAF-CM) were used as fold changes.

### Immunoblotting

Cells were lyzed in RIPA buffer containing phosphatase inhibitor cocktail I (Sigma) and protease inhibitor cocktail mini-tablet (Roche Diagnostics, Indianapolis, IN). The proteins in the lysates (20 μg) were separated by SDS-PAGE and transferred onto polyvinylidenedifluoride (PVDF) membrane (Millipore, Billerica, MA, USA). After blocking with 5% non-fat milk in PBST, the primary antibodies for E-cadherin (Bioworld Technology, BS1098, Louis Park, MN, USA), Vimentin (Proteintech Group, 10366-1-AP, Chicago, IL, USA), α-SMA (DAKO, M0851, Copenhagen, Denmark), ZEB1 (ProteinTech, 21544-1-AP), ZEB2 (Santa Cruz Biotechnology, sc-271984, Santa Cruz, CA, USA), p-Smad2 (Phospho-Ser467, Signalway Antibody, 11322, College Park, MA, USA), Smad2 (Bioworld Technology, AP0444) and β-actin (AbMax, 05–0079, Beijing, China) were used. The membranes were then washed with PBST three times and incubated with horseradish peroxidase (HRP)-conjugated secondary antibody. The Western blots were visualized using the enhanced chemiluminescence reagents (Millipore, WBKLS0100). Western blots were semi-quantified by Image J software (NIH, USA).

### Immunofluorescence

Cells were seeded on the coverslips in 24-well plates and cultured for proper cell density. After fixing by 4% paraformaldehyde for 15 min, cells were washed with PBS, and then blocked with 5% BSA in PBS for 60 min. Primary antibodies targeting E-cadherin (Bioworld Technology), Vimentin (Proteintech) and α-SMA (DAKO) were used overnight at 4 °C. The cells were than washed with PBS and incubated with fluorescein isothiocyanate or phycoerythrin-conjugated secondary antibodies (Cell Signaling Technology, Beverly, MA, USA). DAPI (BBI, D6584, Cambridge, MA, USA) was used for counterstaining. The images were captured by a fluorescence microscope (Olympus DP72, Japan).

### ELISA assay

The concentrations of TGFβ1 in different media were measured using human TGFβ1 ELISA kit (BOSTER, EK0513, Wuhan, China), according to the manufacturer’s instructions. Briefly, after incubation with media for 90 min at 37 °C, the plates were tapped dry and 100 μl biotin labeling TGFβ1 antibody were added for 60 min at 37 °C. The plates were then washed three times using TBS and 100 μl avidin-biotin-pcroxidasecomplex (ABC) were added. Following incubation on an orbital shaker for 30 min at 37 °C, plates were washed by TBS five times and tetramethylbenzidine (TMB) color-substrate solution was added to each well. After incubation in the dark for 30 min at 37 °C, 100 μl TMB stop buffer was used to stop reaction. Then, the plates were read at 450 nm on a tunable microplate reader (Versa Max, Molecular Devices, Sunnyvale, CA, USA).

### Statistical analysis

Data are presented as mean ± standard deviation (SD) from three independent experiments unless special notification. Paired *t* test was used to analyze the difference of ZEB2NAT level between cancer tissues and corresponding normal tissues. Other differences between two groups were analyzed using Student’s *t* test. *P* value less than 0.05 was considered statistically significant.

## Additional Information

**How to cite this article**: Zhuang, J. *et al.* TGFβ1 secreted by cancer-associated fibroblasts induces epithelial-mesenchymal transition of bladder cancer cells through lncRNA-ZEB2NAT. *Sci. Rep.* **5**, 11924; doi: 10.1038/srep11924 (2015).

## Supplementary Material

Supplementary Information
