# Supplementary material for: TGFβ1 secreted by cancer-associated fibroblasts induces epithelial-mesenchymal transition of bladder cancer cells through lncRNA-ZEB2NAT
Source: Sci Rep. 2015 Jul 8;5:11924. doi: 10.1038/srep11924 (PMC4495469; doi:10.1038/srep11924)
Supplement: Supplementary Information [file srep11924-s1.doc]

**Supplementary Information**

**TGFβ1 secreted by cancer-associated fibroblasts induces epithelial- mesenchymal transition of bladder cancer cells through lncRNA-ZEB2NAT**

**Junlong Zhuang^1,,#^, Qun Lu^1,#^, Bing Shen^2,#^, Xiaojing Huang^3^, Lan Shen^3^, Xi Zheng^1^, Ruimin Huang^4^, Jun Yan^3,*^ , Hongqian Guo^1,5,*^**

^1^ Nanjing Drum Tower Hospital, Nanjing University Medical School, Nanjing, Jiangsu 210008, China,

^2^ Department of Urology, Shanghai First People’s Hospital, Shanghai Jiaotong University, Shanghai, 200080, China

^3^ Model Animal Research Center, MOE Key Laboratory Model Animal for Disease Study, Nanjing University, Nanjing, Jiangsu 210061, China

^4^ Department of Nuclear Medicine, Ruijin Hospital, Shanghai Jiao Tong University School of Medicine, Shanghai 200025, China

^5^ Nanjing Urology Research Center, Nanjing, Jiangsu 210008, China

# These authors contributed equally to this work.

*Jun Yan, Ph.D. 12 Xuefu Road, Nanjing, Jiangsu 210061, China. Tel: +86-25-58641535; Email: [yanjun@nicemice.cn](mailto:yanjun@nicemice.cn) and Hongqian Guo, M.D. 321 Zhongshan Road, Nanjing, Jiangsu 210008, China. Tel:+86-25-83105107; Email: dr.ghq@163.com.

**Supplementary Figure 1**

**
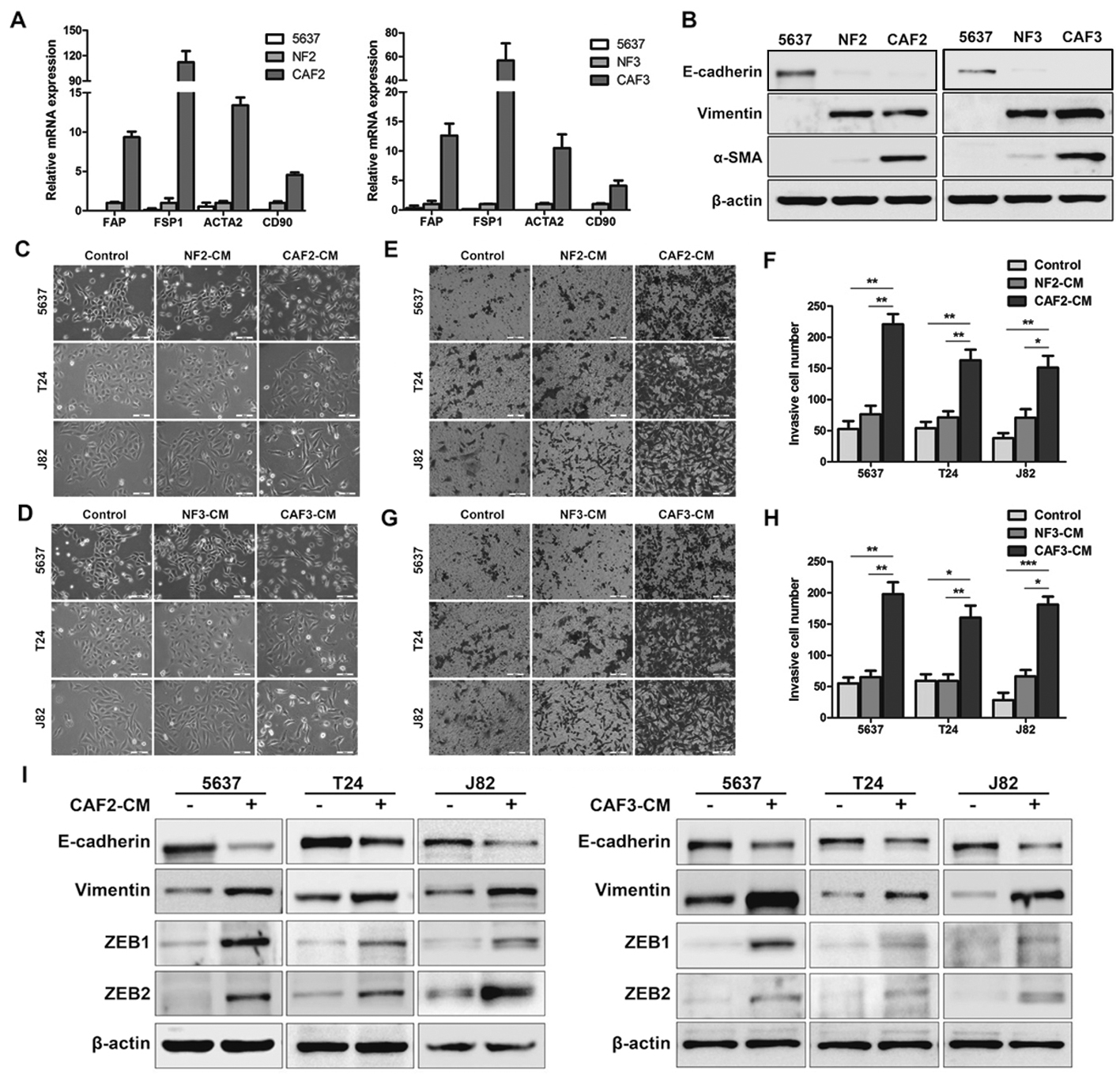
**

**Supplementary Figure 1**. Conditional media from another two CAFs increased the cell invasion capabilities and EMT phenotypes in three bladder cancer cell lines. (A) The mRNA expression levels of CAF-specific genes, including FAP, FSP1, ACTA2 and CD90, in 5637 cells (an epithelial cell control), NFs and CAFs by qRT-PCR using β-actin gene as the normalization control. (B) The protein expression levels of E-Cadherein, Vimentin and α-SMA in 5637 cells, NFs and CAFs were detected by immunoblotting. (C, D) Morphological features of bladder cancer cell lines under culture medium from another two pairs of NF/CAF, respectively. (E-H) Cell invasion was measured by the Transwell cell invasion assay, with quantification. (I) The protein expression levels of E-cadherin, Vimentin, ZEB1 and ZEB2 in the CAF-CM treated bladder cancer cell lines by immunoblotting. β-actin protein was used as the loading control. * *P* < 0.05, ** *P* < 0.01, ****P* < 0.001.

**Supplementary Figure 2**


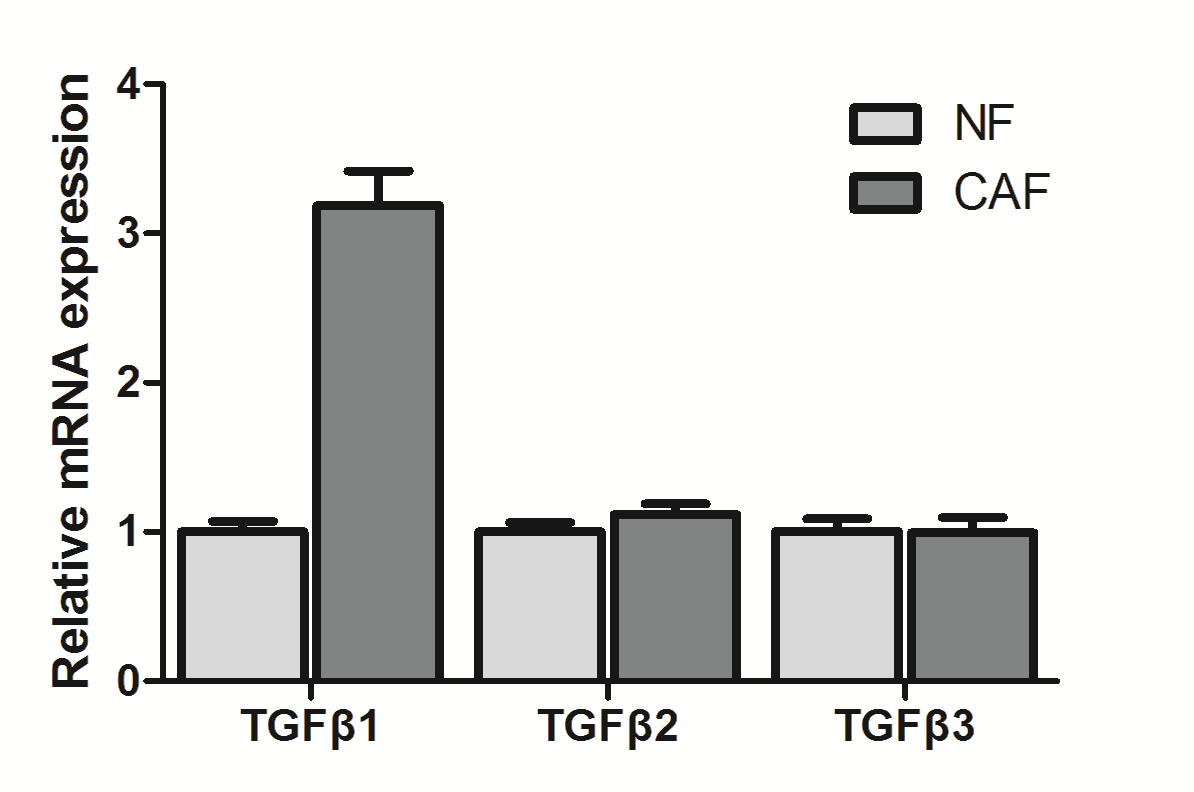


**Supplementary Figure 2**. Three members (TGFβ1, TGFβ2 and TGFβ3) of TGFβ family weredetected in NFs and CAFs by qRT-PCR. CAFs had a 3.2-fold higher expression of TGFβ1 than NFs, but the expression levels of TGFβ2 and TGFβ3 was not significantly changed.

**Supplementary Figure 3**

**
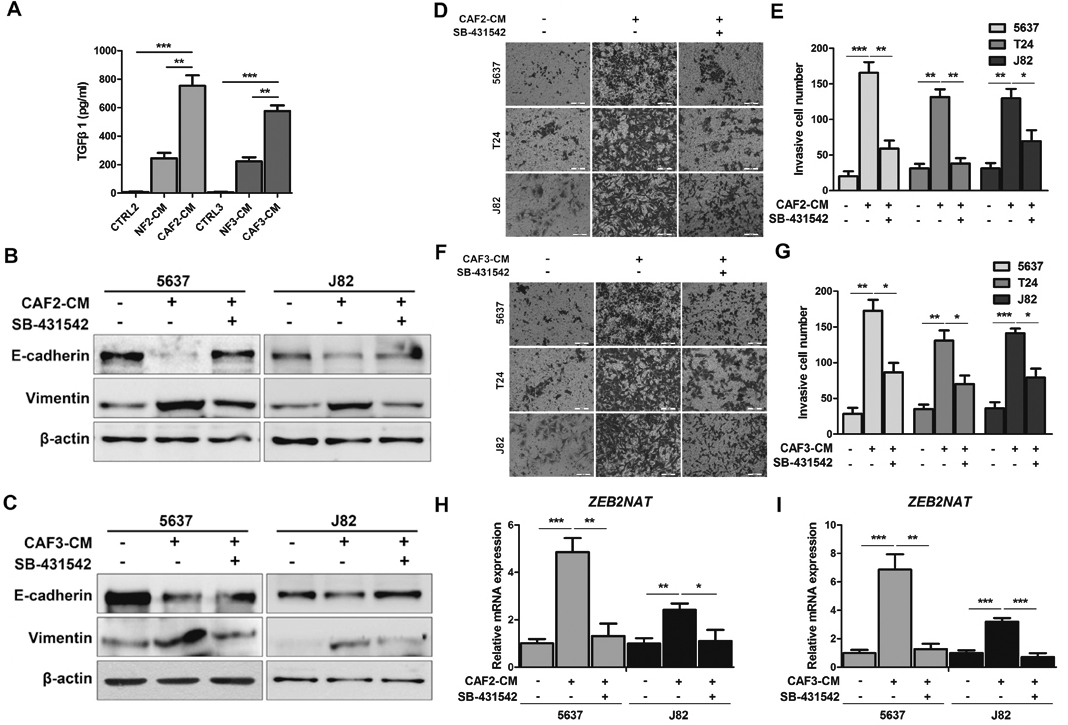
**

**Supplementary Figure 3**. EMT phenotypes were reversed by blocking TGFβ/Smad signaling in the other two CAFs-CM treated bladder cancer cells. (A) TGFβ1 in conditional mediums secreted by 5637 (CTRL), NF and CAF cells were quantified by ELISA. (B, C) The protein levels of E-cadherin and Vimentin in the CAFs-CM treated 5637 and J82 cells upon a TGFβR1 inhibitor (SB-431542) by immunoblotting. (D, E, F, G) Cell invasion ability in the CAFs-CM treated 5637, T24 and J82 cells upon upon a TGFβR1 inhibitor (SB-431542) by the Transwell cell invasion assay. (H, I) ZEB2NAT expression levels in the CAP-CM treated 5637 and J82 cells upon a TGFβRI inhibitor (SB-431542). * *P* < 0.05, ** *P* < 0.01, ****P* < 0.001.

**Supplementary Figure 4**

**
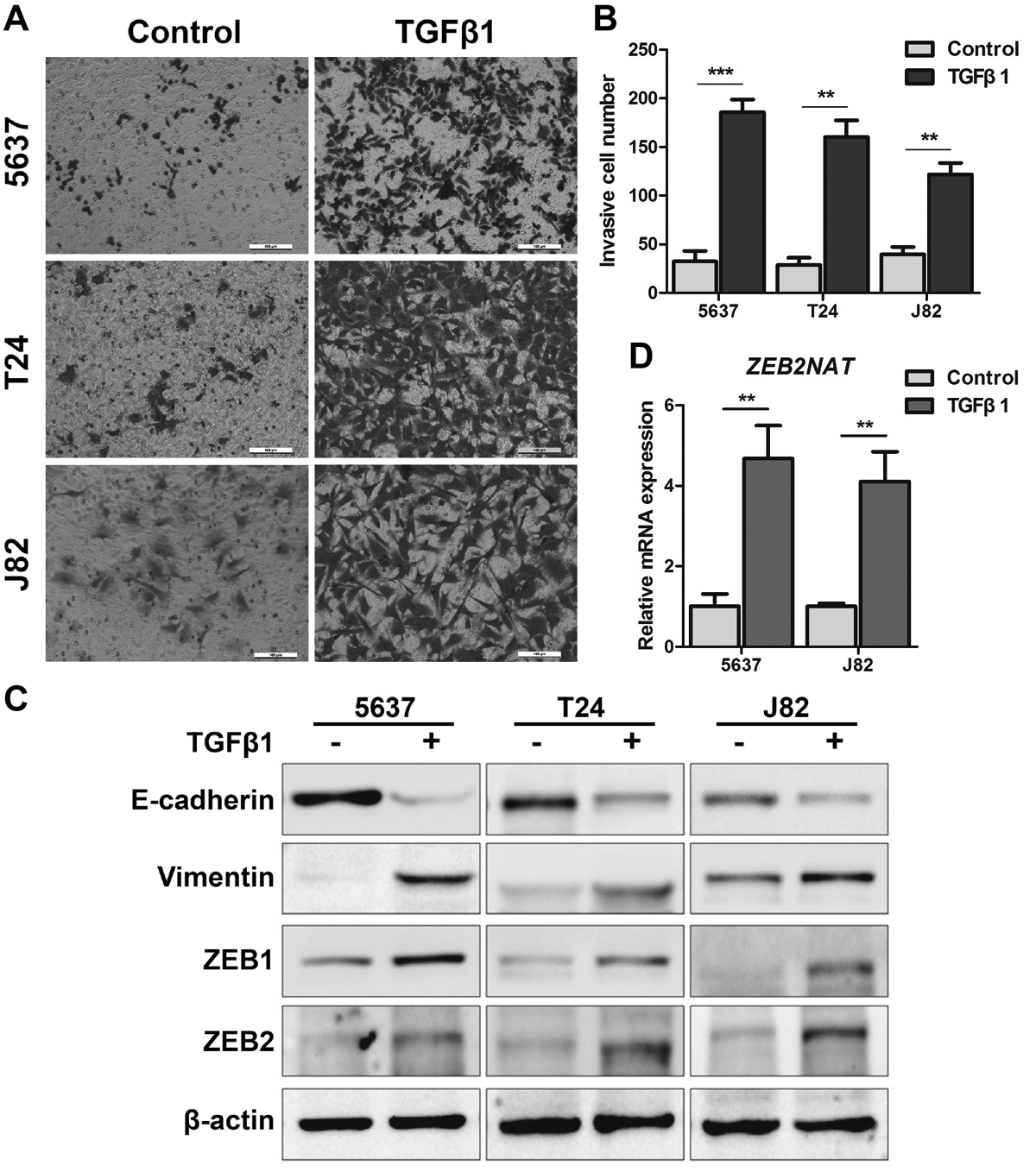
**

**Supplementary Figure 4**. TGFβ1 induced cell invasion and EMT phenotypes in three bladder cancer cell lines. (A, B) Cell invasion was measured by the Transwell cell invasion assay. (C) The protein expression levels of E-cadherin, Vimentin, ZEB1 and ZEB2 in the TGFβ1 treated bladder cancer cell lines by immunoblotting. β-actin protein was used as the loading control. (D) The expression of ZEB2NAT lncRNA in 5637 and J82 cells, detected by qRT-PCR using β-actin gene as the normalization control. ** *P* < 0.01, ****P* < 0.001.

**Supplementary Figure 5**

**
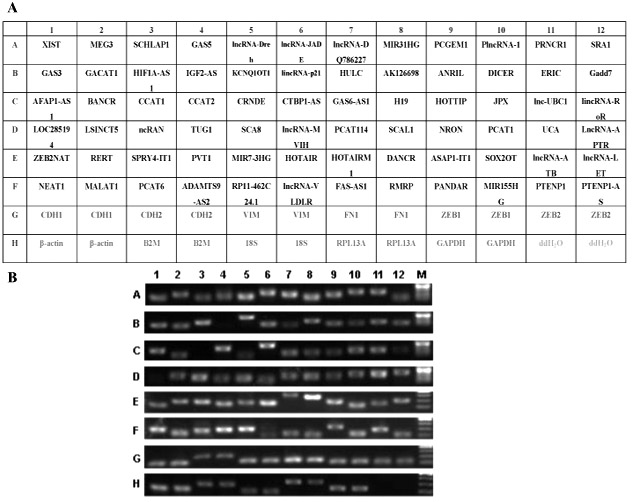
**

**Supplementary Figure 5**. The validation of Human lncRNA Discover PCR array. (A) A layout of the PCR array showing 72 lncRNAs were selected from lncRNA database ([www.lncRNAdb.org](http://www.lncRNAdb.org/)). Besides, there are 6 coding RNAs (in purple) involved in EMT process and 5 house-keeping genes (in blue) as internal controls. ddH_2_O (in green) was used as negative control. (B) Agarose image demonstrated a single band from the qRT-PCR product from cancer cell lines.

**Supplementary Figure 6**


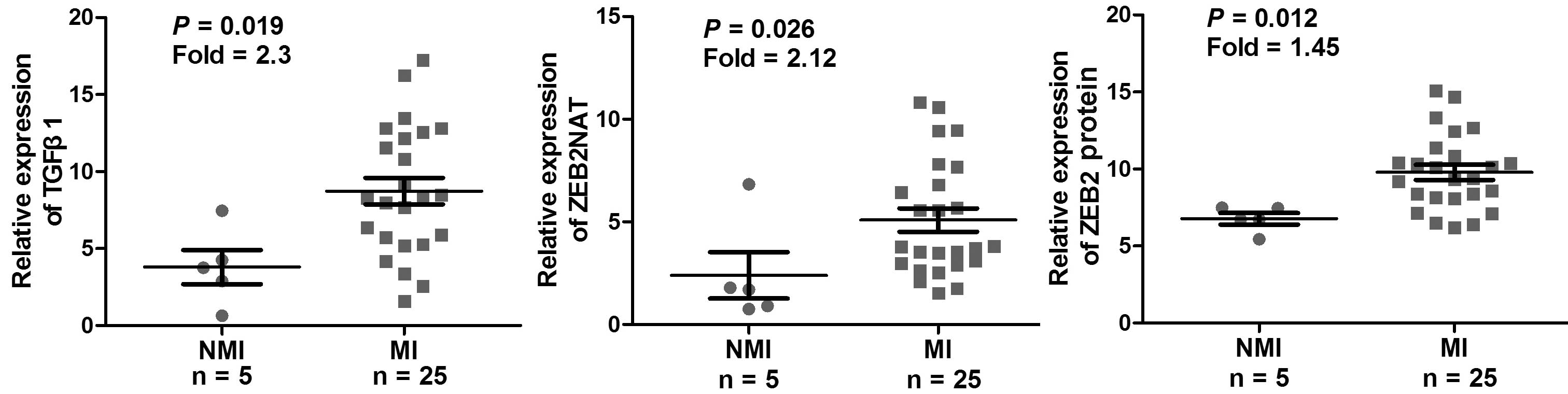


**Supplementary Figure 6.** Overexpression of TGFβ1 and ZEB2NAT transcripts, as well as ZEB2 protein in human muscle invasive bladder cancer samples (MI, n = 25), compared with those in non-muscle invasive bladder cancer samples (NMI, n = 5).

**SUPPLEMENTARY TABLES**

**Table S1. Fold changes of genes in 5637 and J82 cells treated with CAF-CM, compared to NF-CM treatment.**

| **5637** | |  | **J82** | |
| --- | --- | --- | --- | --- |
| ***lncRNA*** | **Folds** |  | ***lncRNA*** | **Folds** |
| lncRNA-VLDLR | 16.6771 |  | lnc-UBC1 | 10.7569 |
| PCAT6 | 6.528658 |  | PCAT114 | 4.16933 |
| ADAMTS9-AS2 | 3.971255 |  | **SPRY4-IT1** | 4.060381 |
| **ZEB2NAT** | 3.601962 |  | UCA | 2.964534 |
| lincRNA-p21 | 3.176559 |  | MIR31HG | 2.827954 |
| HOTAIR | 3.076263 |  | **ZEB2NAT** | 2.430949 |
| **SPRY4-IT1** | 2.926977 |  | **lncRNA-ATB** | 1.607747 |
| SCA8 | 2.907523 |  | CTBP1-AS | 1.598705 |
| PCGEM1 | 2.870073 |  | MIR7-3HG | 1.552689 |
| **lncRNA-ATB** | 2.83072 |  | MIR155HG | 1.432138 |
| XIST | 2.788224 |  | SCAL1 | 1.326566 |
| HIF1A-AS1 | 2.630174 |  | NEAT1 | 1.313308 |
| LOC285194 | 2.477923 |  | SCA8 | 1.261538 |
| CCAT2 | 2.372186 |  | CCAT2 | 1.190457 |
| BANCR | 2.205088 |  | PCAT6 | 1.176841 |
| lncRNA-Dreh | 2.008356 |  | PLncRNA-1 | 1.163489 |
| PCAT114 | 1.428668 |  | HIF1A-AS1 | 1.159122 |
| PRNCR1 | 1.343942 |  | DANCR | 1.149867 |
| RMRP | 1.33931 |  | BANCR | 1.113251 |
| lncRNA-APTR | 1.240318 |  | lncRNA-APTR | 1.101282 |
| GAS5 | 1.206409 |  | FAS-AS1 | 1.092834 |
| lnc-UBC1 | 1.145712 |  | PTENP1 | 1.088984 |
| AFAP10AS1 | 1.114403 |  | HOTAIR | 1.083117 |
| NEAT1 | 1.091484 |  | ASAP1-IT1 | 1.079768 |
| MIR155HG | 1.071975 |  | lncRNA-LET | 1.043395 |
| CCAT1 | 1.063744 |  | AFAP10AS1 | 1.020965 |
| FAS-AS1 | 1.062815 |  | lncRNA-JADE | 0.975452 |
| RP11-462C24.1 | 0.9797 |  | MALAT1 | 0.936953 |
| MIR7-3HG | 0.925877 |  | ADAMTS9-AS2 | 0.93211 |
| GAS3 | 0.907322 |  | lincRNA-p21 | 0.900855 |
| MIR31HG | 0.890108 |  | HOTAIRM1 | 0.89258 |
| PTENP1-AS | 0.888894 |  | Gadd7 | 0.886155 |
| RERT | 0.856972 |  | RMRP | 0.867916 |
| Gadd7 | 0.828894 |  | DICER | 0.856152 |
| CRNDE | 0.828261 |  | PVT1 | 0.85032 |
| SRA1 | 0.810399 |  | ERIC | 0.847109 |
| UCA | 0.810389 |  | lncRNA-MVIH | 0.81685 |
| HULC | 0.79057 |  | XIST | 0.810822 |
| SCAL1 | 0.764707 |  | ANRIL | 0.771078 |
| GACTA1 | 0.763327 |  | ncRAN | 0.770023 |
| ncRAN | 0.759808 |  | SOX2OT | 0.762598 |
| AK126698 | 0.711415 |  | AK126698 | 0.759797 |
| ASAP1-IT1 | 0.695156 |  | GAS5 | 0.758656 |
| SCHLAP1 | 0.677989 |  | JPX | 0.757504 |
| PVT1 | 0.657782 |  | GAS3 | 0.75561 |
| MEG3 | 0.65623 |  | lncRNA-DQ786227 | 0.7504 |
| PLncRNA-1 | 0.632671 |  | TUG1 | 0.730708 |
| lncRNA-LET | 0.629717 |  | RP11-462C24.1 | 0.669301 |
| lncRNA-JADE | 0.626975 |  | **PANDAR** | 0.625513 |
| JPX | 0.618567 |  | **PCAT1** | 0.621121 |
| DANCR | 0.596846 |  | CRNDE | 0.619388 |
| CTBP1-AS | 0.586412 |  | **KCNQ1OT1** | 0.594647 |
| DICER | 0.582721 |  | **NRON** | 0.570825 |
| **NRON** | 0.579821 |  | lncRNA-VLDLR | 0.537089 |
| ANRIL | 0.562731 |  | PTENP1-AS | 0.529384 |
| MALAT1 | 0.543407 |  | HULC | 0.524682 |
| ERIC | 0.529908 |  | SRA1 | 0.521118 |
| TUG1 | 0.501667 |  | lincRNA-RoR | 0.476934 |
| lncRNA-DQ786227 | 0.478819 |  | **HOTTIP** | 0.396829 |
| PTENP1 | 0.449175 |  | PCGEM1 | 0.390606 |
| GAS6-AS1 | 0.445014 |  | PRNCR1 | 0.382526 |
| **HOTTIP** | 0.406624 |  | RERT | 0.369119 |
| lncRNA-MVIH | 0.375183 |  | **H19** | 0.349539 |
| HOTAIRM1 | 0.359725 |  | lncRNA-Dreh | 0.201617 |
| **PANDAR** | 0.355931 |  | LOC285194 | 0.10919 |
| **KCNQ1OT1** | 0.325932 |  | LSINCT5 | 0.100606 |
| SOX2OT | 0.206118 |  | GACTA1 | 0.042344 |
| **PCAT1** | 0.199245 |  | MEG3 | N.D. |
| **H19** | 0.120635 |  | SCHLAP1 | N.D. |
| IGF2-AS | 0.045692 |  | IGF2-AS | N.D. |
| lincRNA-RoR | N.D. |  | CCAT1 | N.D. |
| LSINCT5 | N.D. |  | GAS6-AS1 | N.D. |
| ***mRNA*** |  |  | ***mRNA*** |  |
| CDH1 | 0.530465 |  | CDH1 | 0.630465 |
| CDH2 | 1.293729 |  | CDH2 | 1.261102 |
| VIM | 4.284903 |  | VIM | 1.384295 |
| FN1 | 1.637149 |  | FN1 | 4.336183 |
| ZEB1 | 1.808373 |  | ZEB1 | 1.417923 |
| ZEB2 | 1.845959 |  | ZEB2 | 1.664331 |

**Notes**: genes in red are up-regulated genes, whereas genes in blue are down-regulated genes. N.D. means “not detected”.

**Table S2.** Primers for qRT-PCR:

| **Gene** | **Direction** | **Sequences** | **Gene** | **Direction** | **Sequences** |
| --- | --- | --- | --- | --- | --- |
| **FAP** | Forward (5’-3’) | ATGAGCTTCCTCGTCCAATTCA | **SNAI1** | Forward (5’-3’) | TGCGTCTGCGGAACCTG |
|  | Reverse (5’-3’) | AGACCACCAGAGAGCATATTTTG |  | Reverse (5’-3’) | GGACTCTTGGTGCTTGTGGA |
| **FSP1** | Forward (5’-3’) | GATGAGCAACTTGGACAGCAA | **SNAI2** | Reverse (5’-3’) | CGAACTGGACACACATACAGTG |
|  | Reverse (5’-3’) | CTGGGCTGCTTATCTGGGAAG |  | Forward (5’-3’) | CTGAGGATCTCTGGTTGTGGT |
| **ACTA2** | Forward (5’-3’) | GTGTTGCCCCTGAAGAGCAT | **TWIST1** | Reverse (5’-3’) | GTCCGCAGTCTTACGAGGAG |
|  | Reverse (5’-3’) | GCTGGGACATTGAAAGTCTCA |  | Forward (5’-3’) | TGGAGGACCTGGTAGAGGAA |
| **CD90** | Forward (5’-3’) | ATCGCTCTCCTGCTAACAGTC | **ZEB1** | Reverse (5’-3’) | ACTCTGATTCTACACCGC |
|  | Reverse (5’-3’) | CTCGTACTGGATGGGTGAACT |  | Forward (5’-3’) | TGTCACATTGATAGGGCTT |
| **MMP2** | Forward (5’-3’) | CCGTCGCCCATCATCAAGTT | **ZEB2** | Reverse (5’-3’) | CAAGAGGCGCAAACAAGCC |
|  | Reverse (5’-3’) | CTGTCTGGGGCAGTCCAAAG |  | Reverse (5’-3’) | GGTTGGCAATACCGTCATCC |
| **MMP9** | Forward (5’-3’) | TGGCAGAGATGCGTGGAGA | **TGFβ1** | Forward (5’-3’) | CTAATGGTGGAAACCCACAACG |
|  | Reverse (5’-3’) | GGCAAGTCTTCCGAGTAGTTTT |  | Reverse (5’-3’) | TATCGCCAGGAATTGTTGCTG |
| **CDH1** | Forward (5’-3’) | CACCACGTACAAGGGTCAGGTGC | **TGFβ2** | Forward (5’-3’) | CAGCACACTCGATATGGACCA |
|  | Reverse (5’-3’) | CAGCCTCCCACGCTGGGGTAT |  | Reverse (5’-3’) | CCTCGGGCTCAGGATAGTCT |
| **VIM** | Forward (5’-3’) | ACCAACGACAAAGCCCGCGT | **TGFβ3** | Forward (5’-3’) | ACTTGCACCACCTTGGACTTC |
|  | Reverse (5’-3’) | CAGAGACGCATTGTCAACATCCTGT |  | Reverse (5’-3’) | GGTCATCACCGTTGGCTCA |
| **FN1** | Forward (5’-3’) | TGGCACCCCACGCTCAGATACA | **TGFβRII** | Forward (5’-3’) | GTAGCTCTGATGAGTGCAATGAC |
|  | Reverse (5’-3’) | CTCGCCAGGCAGGTTGACGG |  | Reverse (5’-3’) | CAGATATGGCAACTCCCAGTG |
| **β-actin** | Forward (5’-3’) | CATGTACGTTGCTATCCAGGC |  |  |  |
|  | Reverse (5’-3’) | CTCCTTAATGTCACGCACGA |  |  |  |
